# Supplementary material for: Complexome profiling of the Chlamydomonas psb28 mutant reveals TEF5 as an early PSII assembly factor
Source: Plant Cell. 2025 Mar 17;37(6):koaf055. doi: 10.1093/plcell/koaf055 (PMC12164590; doi:10.1093/plcell/koaf055)
Supplement: koaf055_Supplementary_Data [file koaf055_supplementary_data.zip › psb28_interactive.zip!/plots/intensity/qualityPlots/peptides/_Cre08.g361850.t1.2.html]

### Cre08.g361850.t1.2

Trivial name:   
SeqCoverage [median]: 20.10  
Mass: 25.71 kDa  
  
 

| Sequence | MissedCleavage | Mass [Da] | wtIntensity | muIntensity | Score |
| --- | --- | --- | --- | --- | --- |
| ELFSEQK | 0 | 879.43 | 6713.71 | 6431.75 | 122.15 |
| ITSPEGK | 0 | 730.39 | 3607.30 | 2221.58 | 108.92 |
| ITSPEGKELFSEQK | 1 | 1591.81 | 2739.65 | 2371.69 | 62.29 |
| LSTAADEGGVSTGR | 0 | 1319.63 | 22320.19 | 28746.67 | 272.10 |
| LSTAADEGGVSTGRYR | 1 | 1638.80 | 0.00 | 480.61 | 39.69 |
| NIVDKLQK | 1 | 956.57 | 1067.72 | 0.00 | 88.94 |
| RITSPEGK | 1 | 886.49 | 312.29 | 0.00 | 63.56 |
| TVVYVAYSTR | 0 | 1157.61 | 8080.71 | 22624.57 | 215.14 |
|  |  |  | 44841.6 | 62876.9 |  |
